# Recognition of face-name associations after errorless and errorful learning: an fMRI study

**DOI:** 10.1186/1471-2202-14-30

**Published:** 2013-03-13

**Authors:** Anke Hammer, Claus Tempelmann, Thomas F Münte

**Affiliations:** 1 Department of Neurology, University of Lübeck, Ratzeburger Allee 160, Lübeck, 23538, Germany; 2 Department of Neurology, Otto-von-Guericke University, Leipziger Straße 44, Magdeburg, 39120, Germany; 3 Department of Psychiatry and Psychotherapy, Friedrich-Alexander University of Erlangen-Nuremberg, Schwabachanlage 6, Erlangen, 91054, Germany

**Keywords:** Errorless, Errorful, Executive control, Face-name associations, Recognition, Parietal, Prefrontal

## Abstract

**Background:**

Errorless learning has advantages over errorful learning. The erroneous items produced during errorful learning compete with correct items at retrieval resulting in decreased memory performance. This interference is associated with an increased demand on executive monitoring processes. Event-related functional magnetic resonance imaging (fMRI) was used to contrast errorless and errorful learning. Learning mode was manipulated by the number of distractors during learning of face-name associations: in errorless learning only the correct name was introduced. During errorful learning either one incorrect name or two incorrect names were additionally introduced in order to modulate the interference in recognition.

**Results:**

The behavioural results showed an enhanced memory performance after errorless learning. The veridicality of recognition of the face-name associations was reflected in a left lateralized fronto-temporal-parietal network. The different learning modes were associated with modulations in left prefrontal and parietal regions.

**Conclusions:**

Errorless learning enhances memory performance as compared to errorful learning and underpins the known advantages for errorless learning. During memory retrieval different networks are engaged for specific purposes: Recognition of face-name associations engaged a lateralized fronto-temporal-parietal network and executive monitoring processes of memory engaged the left prefrontal and parietal regions.

## Background

In daily life we have to memorize new information on a regular basis. One particularly difficult memory task is to learn the name of a person we meet for the first time. Consider a cocktail party at which you are introduced by the host to several previously unknown people. This may lead to a situation where several names are associated with a particular face and the incorrect names might interfere with the correct name during retrieval (e.g., when you meet one of the persons again at another party). This interfering information needs to be controlled for and the falsely associated information has to be rejected during memory retrieval.

Functional magnetic resonance imaging (fMRI) studies have linked a fronto-temporal network to the encoding of face-name associations, namely the hippocampus, the fusiform gyrus and the inferior frontal cortex [1-4]. Recognition memory on the other hand depends on several mechanisms including the processing of old versus new information (i.e. the old-new effect) associated with an activation of the prefrontal cortex (PFC) and parietal regions [5-8], for a review see [9,10]. Following Yonelinas [11] sub-processes of recognition memory rely on different neural networks as recollection and familiarity are associated with different regions (*recollection:* an anterior medial region in the PFC, a lateral parietal/temporal region, a medial parietal region, the posterior cingulate, the hippocampus; *familiarity:* lateral PFC including the anterior PFC and the DLPFC, a more superior parietal region, precuneus). Additionally, executive processes controlling the retrieval of information are associated with an activation of prefrontal regions [12-14] that might also reactivate the regions of encoding [9,13,15-17]. In a recent fMRI study, Hayes and colleagues [18] investigated the contributions of the medial-temporal lobes, the PFC and parietal regions to the dual-process theory with recollection vs. familiarity (e.g. source vs. item memory) and the strength theory (e.g. high vs. low confidence). They found a dissociation between a right hippocampal region (high confidence during source memory) and bilateral rhinal regions (high confidence during item memory) supporting the dual-process theory [18]. Further, findings within the left PFC showed greater activity for source compared to item memory being consistent with recollection whereas right ventrolateral areas showed low-confidence activity in source and item memory consistent with monitoring processes [18]. Finally, the parietal regions were consistent with strength theory as source and item memory tasks activated dorsal areas during low confidence and ventral areas during high confidence [18]. Hayes et al., [18] placed this dissociation into an ‘attentional account of parietal activation during episodic retrieval’. There is still an on-going debate, which regions are linked to which process during memory formation. The main results as reported above might be summarized but shortened as follows: temporal regions are linked to recollection [11,18] and familiarity [18]; frontal regions are linked to recollection and familiarity [11,18] with a substantial monitoring component [12-14,18]; and parietal regions are linked to the strength of memory influenced by attentional processes [18].

To study the role of executive control processes during memory retrieval we used different encoding modes, errorful and errorless learning (henceforth EF and EL) as firstly described by Terrace [19,20] in discrimination learning with pigeons. The different learning modes were used to manipulate the presence of interfering information at retrieval [21-24]. During EL learning – a rather managed learning mode – only the correct information is introduced and errors are avoided during the learning process reducing later interference at retrieval. In contrast, EF learning resembles the typical trial-and-error approach. During learning, a number of errors are introduced until the correct response is produced. During retrieval these errors are likely to cause interference.

The disadvantage for EF learning is supposed to be based on the increased activation level of incorrect information during the learning phase which leads to interference [24]. After EL learning this interference does not exist (or is considerably diminished) as only the correct stimulus is presented during learning. Baddeley and Wilson [24] proposed, that for example patients with amnesia could not effectively use explicit memory and rather had to rely on implicit memory in both, after EF an EL learning. The basic idea was that implicit memory as compared to explicit memory does not permit the discrimination between erroneous and correct responses as making an erroneous response during learning may reinforce the error later by priming this error. In cases, in which retrieval is based mostly on implicit memory (for example in memory impaired patients); errors are committed because it is not possible to differentiate between correct and erroneous information leading to a worse task performance after EF compared to EL learning. Thus, episodic or explicit memory processes are needed to resolve the interference in EF modus. Several studies showed that humans can benefit from EL learning. Specifically memory impaired patients due to brain injury [24-28], Alzheimer disease [29-31], and schizophrenic patients [32-35] have been found to exhibit an advantage for EL learning (for a critical review see [36]). The benefits of EL-learning have been demonstrated for different materials (word-lists, word-pairs, sentences, face-name associations, see [36]).

In the present investigation we used fMRI to investigate the localization of executive processes during the recognition of face-name associations following EF and EL learning. Here, face-name associations were learned under three different learning conditions. In the errorless modus, a face was presented visually together with the correct name in the auditory domain (EL). Two EF-modes were used, one with only one incorrect competing name and the correct name (EF1) and one with two incorrect competing names and the correct name (EF2). In comparison to other errorful learning paradigms (e.g. [21-24]) the present errorful learning condition was rather passive as the erroneous information was not actively produced by the participant and the induced interference might be slightly weaker. However, as the two EF modes induced differential levels of response conflict during retrieval based on the interference by the erroneous information, the present design allowed us to study the role of conflict monitoring during the retrieval of face-name associations.

So far, there is only one fMRI study investigating the advantages of EL learning and the disadvantages of EF learning in a word stem completion task in patients with diffuse axonal injury in a blocked design [37]. Due to the blocked design, the the findings are insensitive to the correctness of the response. In the control group an increased activation for EF compared to EL was found within the right posterior cingulate gyrus and the left precuneus [37]. In the patient group, there was an increased activation in bilateral precuneus and bilateral inferior parietal lobules [37]. Electrophysiological studies [21,22] contrasting EF and EL learning implemented in a word stem completion task revealed modulations of the error related negativity (ERN), a component emanating from the pMFC, most likely the anterior cingulate cortex (ACC) as shown by ERP source localization studies [38-42] and error related fMRI activity [43-45]. The modulation of the ERN amplitude in relation to memory decisions following EL or EF learning was linked to the activity of an internal monitoring device assessing the activation of two possible decisions, i.e. the veridicality of retrieved memory traces [21] and the likelihood of making an error [22].

The aim of the current event-related fMRI study is to reveal the neural representations during the recognition of face-name associations after EL or EF learning depending on the correctness of response and the three different learning modes. Correctness of response should be reflected by activity in areas previously associated with memory recognition, namely the prefrontal cortex (PFC) and parietal regions [5-8,18], for a review see [9,10] and the control of the retrieval processes reactivating the regions of encoding [9,13,15,16], namely the hippocampus, fusiform gyri and inferior frontal cortex [1-4]. The different learning modes inducing different degrees of interference during recognition should be reflected in regions associated with executive functions of memory recognition, namely the pMFC including the ACC [38,40-45] and the lateral prefrontal cortex as indexed by error monitoring studies [45-50] and studies on the executive control processes during memory recognition [12-14,26,51]. Here, we expected the highest activation for the learning mode with two distractors and a medium activation for the learning mode with one distractor as compared to EL learning.

## Results

### Behavioral results

In Table 1 the behavioral results are summarized. To normalize performance measures the difference between the percentage of correct responses and the percentage of erroneous responses (hit % - error %) was computed. These measurements were entered into an ANOVA with the three-level factor Learning Mode (EL, EF1, EF2). Performance was modulated by Learning mode (F(2,38) = 3.55, p < .05, Greenhouse-Geisser corrected) with decreasing performance measures from EL over EF1 to EF2. Pairwise comparison resulted in a Bonferroni corrected difference between EF2 and EL (16.4, p < .05). The differences between EF1 and EL (11.0, p = .17) or EF2 and EF1 (5.4, p = 1) did not reach significance.

**Table 1:** Summary of performance measures

	**Hit (SE)**	**Error (SE)**	**Hit (%) – Error (%) *(SE)***
**Performance (%)**			
Errorless	59.9 (2.8)	36.7 (2.3)	23.2 (5.3)
Errorful 1	54.4 (2.3)	42.3 (2.2)	12.1 (5.0)
Errorful 2	51.6 (3.4)	44.9 (2.9)	6.7 (6.4)
**Reaction time (in ms)**			
Errorless	2092 (53)	2663 (96)	
Errorful 1	2086 (56)	2670 (94)	
Errorful 2	2079 (49)	2669 (82)	

The mean reaction times (summarized in Table 1) were subjected into an ANOVA with the factors *Response* (erroneous, correct) and *Learning Mode* (EL, EF1, EF2). There were no response-time differences between the three learning modes but reaction times of erroneous compared to correct responses were clearly delayed. This was mirrored by the statistical analysis revealing a significant main effect for *Response* (F(1, 19) = 81.4, p < .001) whereas the main effect for *Learning Mode* (F(2,38) = 0.02, p > .9) and the interaction *Learning Mode* x *Response* (F(2,38) = 0.10, p > .9) failed to reach significance.

### Neuroimaging data

**Figure 1:**
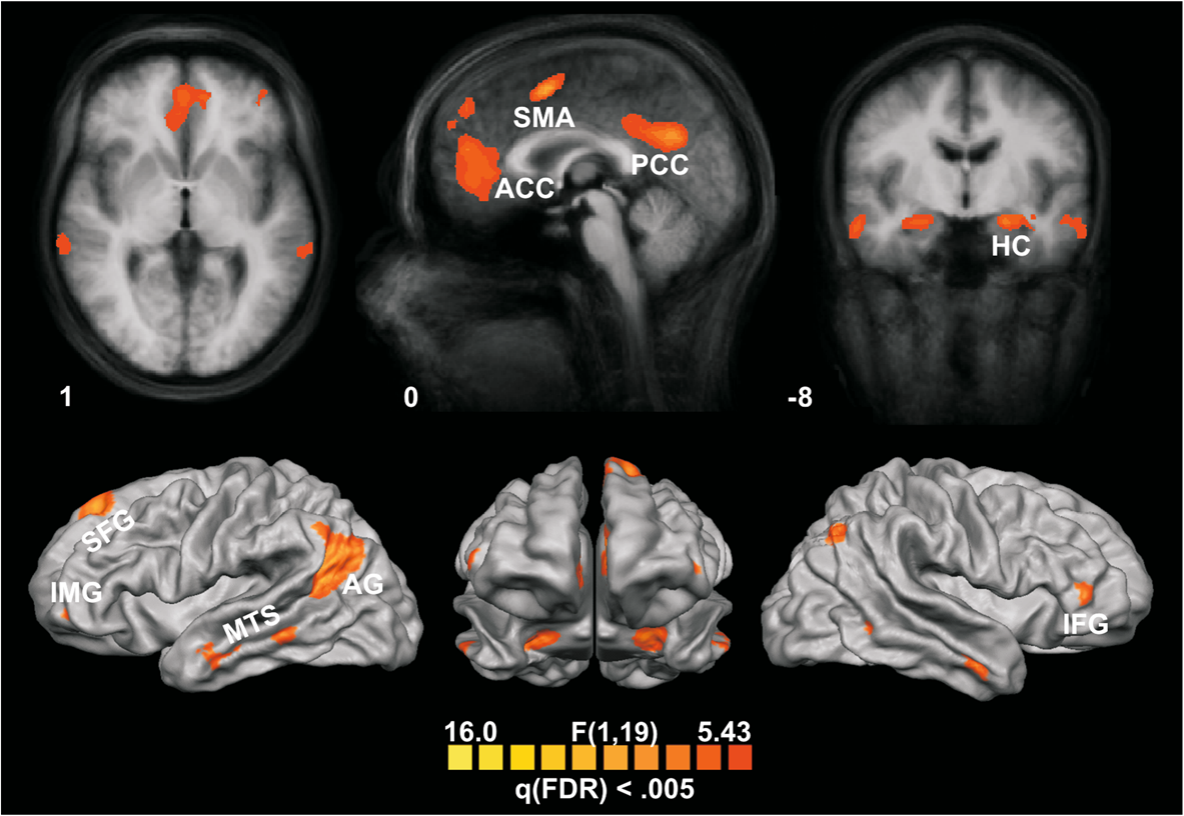
**Cortical statistical maps as revealed by the full ANOVA analysis.** Details of activated regions are given in Table 2. For the corresponding signal changes see Figure 2.

**Figure 2:**
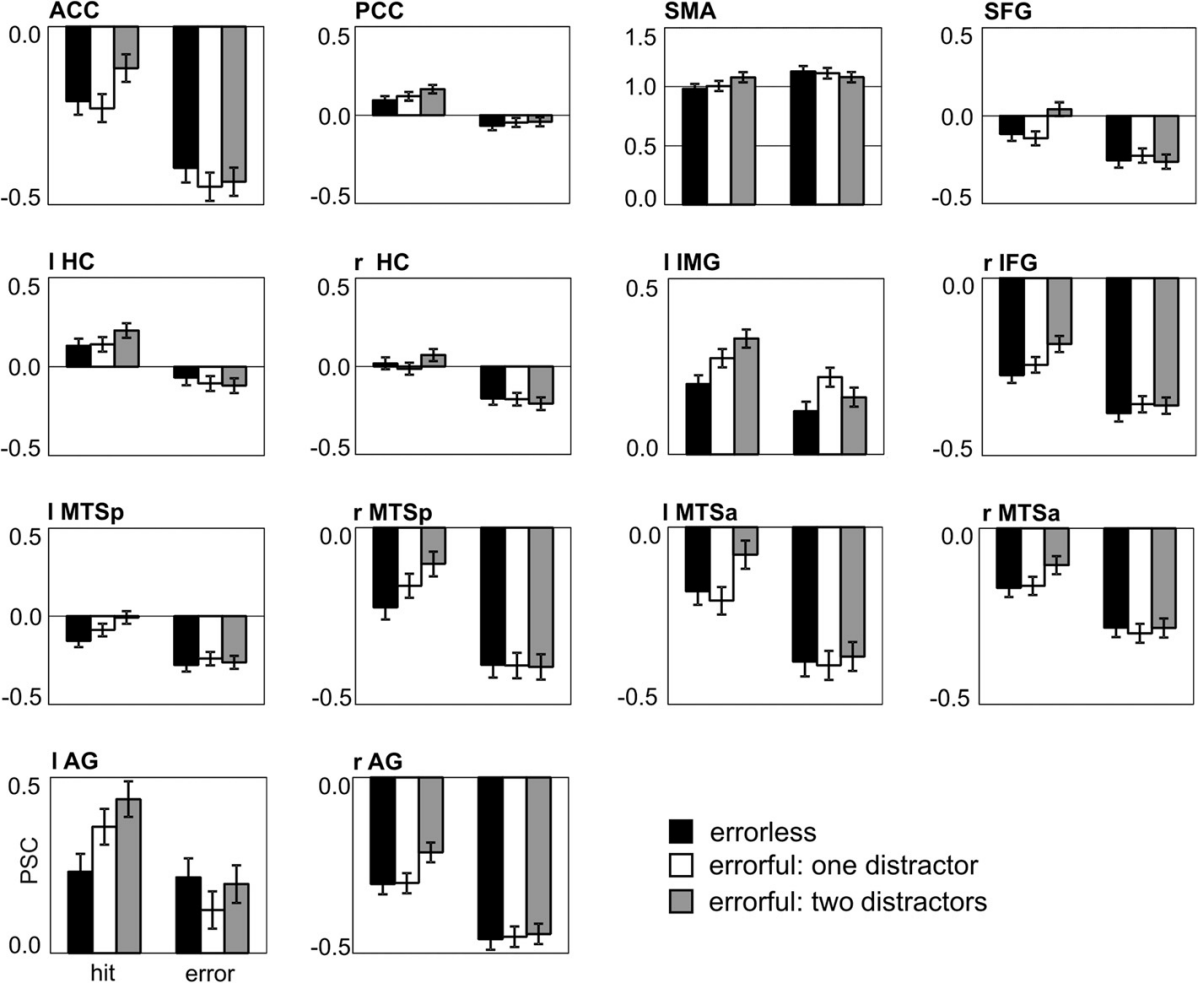
**Diagrams show the mean percentage signal change (PSC) after onset of the face-name association.** Error bars indicate the SE.

The analysis revealed a left lateralized fronto-temporal-parietal network (anterior cingulate cortex (ACC), posterior cingulate cortex (PPC), supplementary motor area (SMA), hippocampus (HC) bilaterally, medial temporal sulcus (MTS) bilaterally, left inferior medial gyrus (IMG), right inferior frontal gyrus (IFG), left superior frontal gyrus (SFG), angular gyrus (AG) bilaterally; see Figure 1 and Table 2). The BOLD responses from these regions quantified as mean percent signal change (PSC) were subjected to ANOVAs including the factors *Response* (correct, erroneous face-name association) and *Learning mode* (EL, EF1, EF2) and their interaction within the region of interest (see Table 3 for statistics and Figure 2 for bar graphs of the PSC). All but one region were sensitive to the correctness of the response as mirrored by the main effect of *Response.* The left IMG showed a trend but failed to reach significance. However, within the left IMG we found a significant learning mode effect and an interaction between Learning Mode and Response. Modulations of Learning Mode were also found for the SFG and the AG bilaterally. Pairwise comparisons are shown in Table 4.

**Table 2:** Results of the overall analysis

**Name of ROI**	**BA**	**Size**	**Talairach**		
		**Nr. of voxel**	**x**	**y**	**z**
1. ACC	32	8968	-1	44	10
2. PCC	23	8431	-1	-50	30
3. SMA	6	3144	-2	7	52
4. l HC	--	2241	-26	-8	-13
5. r HC	--	1763	24	-5	-15
6. l MTSa	21	900	-53	8	-19
7. r MTSa	21	780	58	-7	-19
8. l MTSp	21	963	-62	-36	-3
9. r MTSp	21	239	66	-30	2
10. l IMG	46	87	-40	49	3
11. r IFG	45	622	53	36	13
12. SFG	9	3183	-11	41	48
13. l AG	39	5542	-46	-58	26
14. r AG	39	835	42	-62	35

All reported regions are listed here and provided with presumed Brodmann areas (BA); voxel size; Talairach coordinates as defined by Talairach and Tournoux (1998; x: left-right; y: anterior-posterior origin in anterior commisure; z: inferior-superior). Abbreviations: ROI: region of interest, ACC: anterior cingulate cortex, PCC: posterior cingulate cortex; SMA: supplementary motor area; HC: hippocampus; MTS: medial temporal sulcus; IMG: inferior medial gyrus; IFG: inferior frontal gyrus; SFG: superior frontal gyrus; AG: angular gyrus; l: left; r: right; a: anterior; p: posterior.

**Table 3:** Summary of region of interest analysis

	**Response**	**p**	**LM**	**p**	**Response x LM**	
	**F (1,19)**		**F (2,38)**		**F (2,38)**	**p**
1. ACC	17.49	**.005**	2.18	.1	0.32	.7
2. PCC	20.20	**.001**	2.60	.09	0.27	.8
3. SMA	28.28	**.001**	0.78	.5	0.68	.5
4. left HC	27.72	**.001**	0.21	.8	1.00	.4
5. right HC	36.32	**.001**	0.51	.6	1.22	.3
6. l MTSa	15.79	**.005**	1.39	.3	2.00	.2
7. r MTSa	17.72	**.001**	1.06	.4	0.07	.9
8. l MTSp	17.75	**.001**	3.18	.06	0.45	.6
9. r MTSp	21.74	**.001**	0.65	.45	0.46	.6
10. l IMG	4.02	.06	5.82	**.007**	7.97	**.003**
11. r IFG	24.33	**.001**	1.08	.4	1.27	.3
12. SFG	15.14	**.005**	4.64	**.02**	1.90	.2
13. left AG	20.41	**.001**	3.93	**.03**	0.72	.5
14. right AG	11.03	**.005**	9.09	**.005**	1.12	.4

Abbreviations: LM: learning mode; ACC: anterior cingulate cortex, PCC: posterior cingulate cortex; SMA: supplementary motor area; HC: hippocampus; MTS: medial temporal sulcus; IMG: inferior medial gyrus; IFG: inferior frontal gyrus; SFG: superior frontal gyrus; AG: angular gyrus; l: left; r: right; a: anterior; p: posterior.

**Table 4:** Direct comparison

	**Learning modus (error > hit)**					
	**Errorless**		**Errorful 1**		**Errorful 2**	
	t(19)	p <	t(19)	p <	t(19)	p <
1. ACC	-2.14	**.05**	-3.91	**.001**	-3.60	**.002**
2. PCC	-2.74	**.02**	-3.29	**.004**	-3.70	**.002**
3. SMA	4.44	**.001**	5.57	**.001**	-0.14	.9
4. left HC	-2.74	**.02**	-3.73	**.002**	-4.08	**.001**
5. right HC	-4.37	**.001**	-2.81	**.02**	-3.77	**.002**
6. l MTSa	-1.92	.07	-1.87	.08	-5.42	**.001**
7. r MTSa	-2.80	**.02**	-3.15	**.006**	-3.21	**.006**
8. l MTSp	-2.35	**.03**	-3.07	**.007**	-3.61	**.002**
9. r MTSp	-2.28	**.04**	-3.73	**.002**	-5.29	**.001**
10. l IMG	-0.79	.5	-2.72	**.02**	-2.96	**.009**
11. r IFG	-3.16	**.006**	-3.28	**.004**	-3.61	**.002**
12. SFG	-2.62	**.02**	-1.79	.09	-4.02	**.001**
13. l AG	-2.24	**.04**	-2.74	**.02**	-4.55	**.001**
14. r AG	-1.84	.09	-1.21	.3	-4.10	**.001**
	**Erroneous answers**					
	**EF2 > EL**		**EF1 > EL**		**EF2 > EF1**	
	t(19)	p <	t(19)	p <	t(19)	p <
10. l IMG	-0.11	1	2.10	**.05**	-1.92	.07
12. SFG	1.31	.3	1.50	.2	0.41	.7
13. l AG	0.86	.4	0.99	.4	0.19	.9
14. r AG	1.00	.4	1.11	.4	-1.34	.2
	**Correct answers**					
	**EF2 > EL**		**EF1 > EL**		**EF2 > EF1**	
	t(19)	p <	t(19)	p <	t(19)	p <
10. l IMG	4.20	**.001**	1.5	.2	3.03	**.007**
12. SFG	2.46	**.03**	-0.75	.5	3.14	**.006**
13. l AG	2.33	**.04**	0.63	.6	1.90	.08
14. r AG	3.17	**.006**	2.08	.06	2.17	**.05**

Abbreviations: EL: errorless; EF1: errorful one distractor; EF2: errorful 2 distractors; ACC: anterior cingulate cortex, PCC: posterior cingulate cortex; SMA: supplementary motor area; HC: hippocampus; MTS: medial temporal sulcus; IMG: inferior medial gyrus; IFG: inferior frontal gyrus; SFG: superior frontal gyrus; AG: angular gyrus; l: left; r: right; a: anterior; p: posterior.

Within most regions (ACC, PCC, HC bilaterally, right MTSa, MTSp biltaterally, right IFG, and the left AG), the correctly identified face-name associations showed an increased activity as compared to the erroneous face-name associations. Differences in activity between erroneous and correct face-name associations for the errorless condition were found in the SMA and the SFG, for the errorful condition with one distractor in the SMA and the left IMG, and for the errorful condition with two distractors in all regions but the SMA.

For regions with a Learning Mode effect we directly compared the different learning mode conditions for correct and erroneous face-name associations. Solely within the left IMG a differentiation between erroneous face-name associations was observed. More specifically, the errorful condition with one distractor was significantly increased as compared to the errorless condition. However, for the correct face-name associations there was increased activity for the errorful condition with two distractors as compared to the errorless condition within the left IMG, the SFG, and the AG bilaterally. No differentiations were found for the errorful condition with one distractor compared to the errorless condition. However, a differentiation between both errorful conditions (EF2 > EF1) was found within the left IMG, the SFG and the right AG.

## Discussion

The aim of the study was to investigate which brain areas are involved in different aspects of the recognition of face-name associations learned under different encoding modes (errorless learning and errorful learning with either one or two distractors). As expected, EL led to superior memory performance as the best performance was found for EL, a medium performance for EF1 and the worst performance for EF2. The advantage of errorless learning over errorful learning has been demonstrated in a number of previous studies [21-24,52] and the present results further support the advantage of errorless learning for face-name associations as compared to errorful learning, specifically for errorful learning with an increased number of distractors. Baddeley and Wilson [24] proposed, that the disadvantage of EF learning is based on the increased activation level of incorrect information during learning which leads to interference. Such interference from multiple activated items is supposed to be disturbing for persons with memory impairment as these rather rely on familiarity decisions. After EF learning, both correct and erroneous information are familiar and only the recollection of specific aspects of the learning episode keep these items apart (see also [21]). According to Rodriguez-Fornells [21], it is the necessity to recollect the learning episode for items learned under EF but not EL conditions that places the EF/EL paradigm into the context of the source-monitoring framework [53,54]. Here, monitoring processes need to be applied to old and new items in order to differentiate between them and can be made explicit by asking participants not only to decide whether a given item is new or old but also make a secondary decision for old items that reveals knowledge about encoding [21]. Memory strength (see e.g. [55]) could be taken into account as an alternative interpretation for the benefit of EL over EF learning: The additional erroneous information during EF learning produced interference and led to a more weakly encoded memory representation for the correct target name at recognition. To confirm this interpretation, memory strength should be assessed by e.g. participants’ confidence ratings in all learning conditions.

The main focus was the neural basis of the executive control mechanisms modulated by the three learning modes. The incorrect information induced during learning interferes with the correct information during retrieval. This interfering information needs to be controlled for and the falsely associated information has to be rejected during memory retrieval. Thus, the most challenging situation was supposed to be recognition after errorful learning with two distractors (EF2) followed by errorful learning with one distractor (EF1) as compared to errorless learning with no distractor (EL) as errors were avoided during errorless learning.

A left lateralized fronto-temporal-parietal network (anterior and posterior cingulate cortex, supplementary motor area, bilateral hippocampus, bilateral medial temporal sulcus, left inferior medial gyrus, right inferior frontal gyrus, left superior frontal gyrus, and bilateral angular gyrus) associated with the recognition of face-name associations after errorless or errorful learning was found. The general pattern of the network is in accordance with earlier findings: Recognition memory was found to activate a prefrontal-parietal network [5-8], for a review see [9-11,56]. A recent fMRI study including differential aspects of memory tasks revealed a network including temporal, prefrontal and parietal regions [18]. The activation of the prefrontal regions are in accordance with the sensitivity of these regions to executive processes controlling retrieval [12-14], which in turn might reactivate the regions involved during encoding [9,13,15-17] as for example the hippocampus and the inferior frontal cortex [1-4]. The activation of frontal regions is supported by previous research using event-related brain potentials to memory decisions have revealed an error-related negativity for memory decisions modulated by EL and EF modes, a component emanating from the pMFC [21,22]. Here, most of the reported regions were driven by correctness (correct > error) without an influence of learning mode, resembling the old-new effect (hits > correct rejections) [5-8], for a review see [9,10]. Specifically, medio-temporal areas including the hippocampus have been reported to be involved in the encoding of new information and the reactivation/re-access of these regions by recognition processes and executive processes [9,11,13,15-17]. As expected, executive control did not modulate temporal regions, which would have been indexed by a modulation of learning mode.

The focus of the current study was to evaluate the regions associated to executive control modulated by the three different learning modes. Following the error monitoring literature we expected to find a differential activation of learning mode within the pMFC including the ACC. However, modulations within the ACC were only driven by the correctness of response indexed by a decrease for errors as compared to correct face-name associations. As learning face-name associations is difficult, the failure to detect a modulation of the pMFC driven by learning mode might be a ground effect. On the other hand, the ACC might be sensitive to the veridicality of the memory decision in difficult memory tasks as one part of the network supporting other regions in further fine-grained processing of monitoring demand. The left inferior frontal region and the angular gyrus seem to be the core regions in monitoring memory processes as these were sensitive to the correctness of response and to learning mode. As already mentioned above, both regions have been found previously in the context of memory recognition paradigms and as expected the left lateral prefrontal region [12-14,26,51] was sensitive to executive processing. The interference in recognition due to the incorrect face-name associations induced during learning was reflected in the activity modulation of the left inferior frontal gyrus: correctly identified faces-name associations which were learned with two incorrect names (high interference, EF2) resulted in an increased activation followed by the faces-name associations which were learned with one incorrect name (medium interference, EF1) and the errorless learned face-name associations (no interference, EL). A similar activation pattern was found for the left angular gyrus, with an increased activation for the face-name associations with high interference (EF2) followed by the medium interference (EF1) and the lowest for the face-name associations with no interference (EL). The data indicates that the left lateralized PFC and part of the parietal cortex are sensitive to executive control of memory processing. This finding is in accordance with earlier reports that the prefrontal and parietal regions have been reported to mediate processes that guide recollection attempts via maintenance and elaboration of retrieval cues and monitoring the products of recollection [12,13,16,57] or a more general executive control system that is involved in the processing of familiarity and recollection [11] possibly influenced by attentional processes dependent on memory strength [18]. However, this possibility needs to be evaluated by confidence.

## Conclusions

Errorless learning enhances memory performance as compared to the trial-and-error approach and underpins the known advantages for errorless learning and the disadvantages for errorful learning, which increases with the amount of interference based on the induced incorrect information during learning phase. Recognition of the face-name associations was associated to a left lateralized fronto-temporal-parietal network. More importantly, executive processes driven by learning mode were reflected in left prefrontal and parietal regions.

## Methods

### Subjects

Twenty German native speakers (11 women, age: 24.2 +/-2.5 years) gave written consent to participate for either course credit or a small monetary compensation. All had normal vision, were right-handed and neurologically healthy. All procedures had been approved by the local ethics committee in Magdeburg.

### Measurements

Functional measurement: BOLD dependent functional magnetic resonance images were obtained using a 3 Tesla Siemens Magnetom Trio Vision system (Siemens, Erlangen) equipped with an eight channel phased array head coil. The functional images were acquired with a gradient echo EPI sequence (TR = 2 s, TE = 30 ms, FOV = 220 × 220 mm^2^, flip angle = 80°, matrix size = 64 × 64, in-plane resolution 3.4375 × 3.4375 mm^2^, slice thickness = 3.5 mm, interslice gap 0.35, 30 slices oriented parallel to the AC-PC-line, specified with a midsagittal scout image). To allow participants to rest in between, three separate functional runs were acquired. One functional run comprised 396 volumes and lasted 13.2 minutes. In order to avoid a T1 saturation effect we did not present any material during the first 7 volumes and excluded the first four volumes from further analyses.

Anatomical measurement: A high-resolution T1 weighted 3D-MPRAGE image was acquired as anatomical reference (TR = 1800 ms, TE = 3.44 ms, flip angle = 7°, FOV = 256 mm, matrix size = 256 × 256, 192 sagittal slices, in-plane resolution 1 × 1 mm^2^, slice thickness = 1 mm).

### Material and design

The session comprised a study and a recognition phase. The study phase was performed outside the scanner, while the recognition phase was performed inside the scanner acquiring the functional scans as described above. In the study phase 72 face-name-associations had to be learned, which were assigned to three different learning conditions (errorless, EL; errorful 1, EF1; errorful 2, EF2). There were 24 faces (12 women and 12 men) per condition. In each trial, an unfamiliar face subtending 3.5° × 5° of visual angle in width/height was presented in black and white for 8 seconds on a video-screen followed by a fixation cross. In addition, names were presented auditorily. In an EL trial, only the correct name associated with the face was presented after 6 seconds (e.g., “This is Peter.”). In an EF1 trial an incorrect name (after 3 seconds) and the correct name (after 6 seconds) associated with the face were presented (e.g., “This is not Klaus. This is Dieter.”). In an EF2 trial, two incorrect (0.3 and 3 seconds) names and the correct name (6 seconds) associated with the face were presented (“This is not Paul. This is not Walter. This is Michael.”). In order to avoid order effects and individual learning strategies, the three learning conditions were presented in a pseudo-randomized order.

In the recognition phase, these faces were presented in a randomized order three times each in order to increase the power of BOLD signal (resulting in 72 stimuli per condition). Each face was presented for 3 seconds with a stimulus onset asynchrony of 4 to 12 seconds with an increment of 2 seconds (i.e. a multiple of a TR). A fixation cross was presented between the stimuli. Three names were shown below each face. The participant had to indicate the correct name by pressing one of three buttons. Faces from the EL condition were accompanied by the correct name and two new incorrect names, which did not occur in the study phase before in conjunction with any face. Faces presented in the EF1 condition were accompanied by the correct name, the incorrect name presented in the study phase and a new incorrect name. In the EF2 condition the correct name and the two incorrect names of the study phase were presented. Each name was only used once. Altogether 108 female and 108 male names were needed. These names were selected from communal statistics of newborn children between the years 1970s and 1980s indicating how often a certain name was assigned to children within that time span. The frequencies of the names were equated over the three conditions. The face name associations were rotated between participants, thus each participant associated different names with different faces.

Each scanning session started with a scout image to obtain position information. Right after that, the functional scans (396 volumes, 72 face-name-associations, 24 associations per condition) were performed followed by the structural scan. The entire experiment lasted about 90 minutes (including instructions, preparation and study and recognition phase).

### Image analysis

Image analysis was performed using BrainVoyager QX software (Brain Innovation B.V., Maastricht, The Netherlands). Prior to data analysis, all images were corrected for motion parameters and slice-scan time order, co-registered with the subjects’ corresponding anatomical (T1-weighted) images, normalized into standard coordinate system (Talairach and Tournoux, 1988), and spatially smoothed using a 8 mm full-width-at-half-maximum Gaussian kernel. Additionally, linear drifts were removed from the signal and data were high-pass filtered to remove slow frequency drifts up to 3 cycles per time course. Furthermore, surface rendering, and cortex reconstruction were performed.

For multiple regression analysis of the functional data, a random effects general linear model (GLM) with predictors for each of the six experimental conditions (correct and erroneous responses to the errorless face-name association (EL), the errorful face-name association with one distractor (EF1), and the errorful face-name association with two distractors EF2) was computed. Trials without responses (overall 3.4%, 1.2 SEM, no differences between conditions, F (2,38) = 1.82, p = .2) were defined as predictors too, but no included in later analysis. Onset times of the regressors (convolved with a two gamma HRF) were the time points of appearance of the face-name association. Fixation periods served as baseline.

We applied a random-effects analysis using single-factor repeated measures ANOVA (RFX ANOVA) including the critical predictors (6 levels: correct EL, EF1, EF2 and erroneous EL, EF1, EF2). Thresholding was controlled by False Discovery Rate (FDR) at 5% and c(V) = 1 [58]. In addition, activated clusters were only accepted if more than 50 voxels were significantly activated. All reported activations are based on group statistics. To assess differences between conditions within regions of interest (ROI; as revealed by the RFX-ANOVA) we performed a 3 × 2 ANOVA crossing the factors Learning mode (EL, EF1, EF2) and Response (correct, erroneous face-name associations). This analysis was followed by planned pair wise comparisons (see result section).

## Competing interest

The authors declare that the research was conducted in the absence of any commercial or financial relationships that could be construed as a potential conflict of interest.

## Authors’ contributions

AH: designed the experiments, performed the acquisition of the data and the statistical analysis and drafted the manuscript. CT: contributed to the acquisition of the data and revised the manuscript critically for important intellectual content. TFM: conceived and designed the experiment and drafted the manuscript. All authors read and approved the final manuscript.
